# AI in medical and dentistry education: perspectives from international students, educators and physicians

**DOI:** 10.1186/s12909-026-08886-5

**Published:** 2026-02-25

**Authors:** Timea Németh, Shaya Irandoust, Anna Dávidovics

**Affiliations:** 1 Department of Languages for Biomedical Purposes and Communication, University of Pécs Medical School, Szigeti str 12, Pécs, 7624 Hungary; 2 Department of Languages for Biomedical Purposes and Communication, AI in Medical and Dentistry Education Research Team, University of Pécs Medical School, Szigeti str 12, Pécs, 7624 Hungary; 3 Department of Biochemistry, Oslo New University College, Ullevålsveien 76, Oslo, 0454 Norway

**Keywords:** Artificial intelligence (AI), Medical education, Dentistry education, International medical and dentistry students, Chatbots, ChatGPT, Generative AI, Ethical guidelines, Curriculum development, Stakeholder perspectives

## Abstract

**Background:**

Artificial intelligence (AI) is being integrated into higher education in an increasing rate, offering opportunities for personalized learning, efficiency, and enhanced engagement. However, most research focuses on single-institution or national contexts and primarily examines medical students through quantitative methods. Little is known about how international medical and dentistry students, educators, and practising physicians perceive and experience AI in medical and dentistry education. This study addresses this gap by capturing the perspectives of these diverse stakeholders, providing a comprehensive view of AI adoption, readiness, and ethical considerations across international contexts.

**Methods:**

This cross-institutional mixed-methods study combined survey and interview data to explore perspectives on AI in medical and dentistry education. An online questionnaire was distributed to medical and dentistry students at the University of Pécs Medical School, Hungary and Oslo New University College, Norway. Semi-structured interviews were conducted with educators (*n* = 6) and physicians (*n* = 3) in October, 2024. Quantitative data were analyzed using descriptive statistics and inferential analyses to examine differences across participant groups and qualitative data were examined thematically and comparatively across educators and practising physicians.

**Results:**

A total of 344 medical and dentistry students completed the survey. The majority of students (75.3%) reported that AI tools enhanced their learning and found chatbots beneficial for assignments (73.5%). AI was viewed as helpful for understanding complex concepts (65.1%) and managing study time (42.1%). However, concerns included data privacy (48.9%), reduced critical thinking (58.7%), and over-reliance on AI (74.7%). Comparative analysis revealed differences in emphasis across stakeholder groups, with students focusing on practical learning benefits and educators and practising physicians prioritizing long-term professional and ethical implications. Across groups, there was consensus that AI should supplement but not replace traditional and clinical teaching.

**Conclusions:**

AI is perceived as a valuable supportive tool in medical and dentistry education, particularly by students, while educators and practising physicians adopt a more cautious stance emphasizing ethical, pedagogical, and clinical considerations. Effective integration of AI requires balanced implementation, including AI literacy for students, targeted faculty development, and clear institutional and ethical frameworks to safeguard critical thinking and professional competencies.

**Supplementary Information:**

The online version contains supplementary material available at 10.1186/s12909-026-08886-5.

## Introduction

Artificial intelligence (AI) has rapidly gained prominence in higher education, with medical and dentistry education increasingly positioned at the forefront of this development. AI-based applications, including chatbots, adaptive learning platforms, and generative tools, are now widely regarded as useful for personalizing learning, simulating clinical reasoning, and providing immediate feedback to students [1]. Such tools are increasingly used in medical and dentistry training, where the volume and complexity of knowledge present unique challenges to both students and educators [2]. The early integration of AI into medical and healthcare education has therefore been described not only as a pedagogical innovation but also as a necessary step to prepare future physicians for practice in AI-enhanced healthcare environments [3].

Empirical research on AI in education has expanded rapidly since the introduction of generative AI platforms such as ChatGPT in late 2022. Most studies, however, focus primarily on students and rely on quantitative surveys. In their study, Issa et al. [3] surveyed 642 health profession students in Jordan, the United Arab Emirates, Saudi Arabia, and Egypt reported that 91% viewed AI positively in healthcare and 77.6% supported its curricular integration, although 66.4% demonstrated limited baseline knowledge of AI concepts. In a Swedish national survey of 5,894 students, 95% reported familiarity with ChatGPT and more than one-third (35%) used it regularly [4]. The same study found that 56% held positive attitudes toward AI chatbots, and nearly half (48%) believed such tools made them more effective learners, although 62% agreed that using chatbots for assignments or examinations constituted cheating. Similarly, Sami et al. [5] examined medical students’ perspectives on AI integration in Pakistan, assessing perceptions of AI’s effectiveness and credibility.

Other studies have highlighted both opportunities and challenges. In India, a cross-sectional survey of 325 medical students revealed that 57.2% believed AI could reduce medical errors and 54.2% thought it would enhance decision accuracy. However, 69.2% were concerned that it might reduce the humanistic aspects of medicine, and more than half expressed apprehension about confidentiality and patient trust [6]. A systematic review of 112 studies further underscored this duality: AI tools such as ChatGPT can enhance engagement, accessibility, and personalized learning, but persistent issues include output inaccuracy, bias, plagiarism risk, and excessive dependence on technology at the expense of critical thinking [1]. Broader higher education analyses have reached similar conclusions, emphasizing both the pedagogical potential of generative AI and the need for ethical frameworks and faculty preparedness [2, 7].

While research on AI in medical education has increased, most published studies remain student-focused and predominantly quantitative in design. These surveys [3–5] provide valuable insights into student familiarity, benefits, and concerns, yet often fail to capture the perspectives of educators and practising physicians, who are critical stakeholders in shaping curricula and ensuring clinical relevance Similar methodological patterns can be observed in broader educational research within medical training. For example, a recent national study in Hungary employed the DREEM framework to examine medical and dental students’ perceptions of their learning environment across multiple universities [8]. While that study offered valuable insights into students’ overall educational experiences, it did not address the emerging technological, ethical, and pedagogical dimensions now introduced by AI. Addressing such complexities requires research that integrates multiple stakeholder perspectives. In this regard, Moldt et al. [9] surveyed stakeholders using qualitative interviews to assess awareness of AI and identify essential competencies for its integration into medical and healthcare education. However, few investigations combine survey data with qualitative insights that contextualize numerical findings, such as educators’ concerns about critical thinking or professional integrity. Reviews similarly note that empirical, multi-stakeholder research remains limited, with most analyses emphasizing conceptual opportunities or student-centred findings [1, 2]. Furthermore, few studies consider the international dimension, leaving gaps in understanding how cultural, institutional, and systemic differences shape perceptions of AI adoption.

This research gap highlights the need for a cross-institutional, mixed-methods study examining how students, educators, and practising physicians perceive AI’s role in medical and dentistry training. Such perspectives are essential for developing balanced approaches that maximize educational benefits while safeguarding clinical reasoning, ethical standards, and professional identity. Therefore, this study aims to explore the experiences, perceptions, and attitudes of medical and dentistry students, educators, and physicians from multiple international backgrounds toward AI in medical education, identifying its strengths, limitations, and potential future directions.

### Research questions

Based on this gap, the study was guided by the following research questions:

- RQ1: What are the current experiences, perceptions, and attitudes of medical and dentistry students, educators, and practising physicians regarding the use of AI in medical and dentistry education?
- RQ2: How do the perspectives of these three stakeholder groups differ, particularly regarding the perceived benefits and risks of AI integration?
- RQ3: What ethical, pedagogical, and practical challenges are associated with the use of AI in medical and dentistry education from a multi-stakeholder perspective?
- RQ4: What recommendations do stakeholders propose for the effective and ethical integration of AI into future medical and dentistry curricula?

### Theoretical framework

This study draws on the Technological Pedagogical Content Knowledge (TPACK) framework [10] and its recent reconceptualization for the era of generative AI [11]. TPACK conceptualizes educators’ knowledge as the intersection of content (CK), pedagogy (PK), and technology (TK). Effective technology integration depends on how these domains interact in context. Mishra et al. [11](2023) propose expanding TPACK to include *Contextual Knowledge (XK)*, which accounts for ethical, societal, and long-term implications of AI. This extension reframes technological knowledge to include not only operational skills but also the capacity to critically evaluate AI outputs, identify bias, and design pedagogical strategies that employ generative AI responsibly. In this study, the extended TPACK model guided the design and analysis of surveys and interviews with medical and dentistry students, educators, and physicians. Participants’ skills, attitudes, and practices were mapped to the TPACK domains and the contextual dimension to examine how generative AI is reshaping professional knowledge in medical and dentistry education.

## Methods

### Study design

This study employed a cross-institutional, mixed-methods design conducted across two higher education institutions: the University of Pécs Medical School (UPMS), Hungary, and Oslo New University College (ONH), Norway. It integrated quantitative survey data, qualitative interview data, and insights from the existing literature. This mixed-methods designs enable triangulation, combining numerical trends with contextual insights to achieve both breadth and depth [12].

### Setting and participants

Participants were recruited from UPMS (Hungary) and ONH (Norway), which are linked through a joint 1 + 5 medical education program. ONH students complete their first year of medical and dentistry studies in Oslo before continuing at UPMS, creating a coherent basis for cross-institutional analysis. The study targeted three stakeholder groups from both institutions: medical and dentistry students, educators actively involved in teaching, and practising physicians affiliated with teaching hospitals. Of approximately 500 invited students at UPMS and 100 at ONH, 344 completed the survey, corresponding to an estimated overall response rate of 57%. Nine interview participants (six educators, three physicians) had 10–20 years of teaching or clinical experience, representing both educational and healthcare settings.

### Quantitative data collection

For the online questionnaire, medical and dentistry students were invited to participate via institutional mailing lists, classroom announcements, social media platforms, and snowball sampling was applied. The online survey was conducted in English using the Google Forms platform between October 1 and October 15, 2024.

Although English is not the primary language of instruction at ONH, students there typically attain high English proficiency from an early age and complete portions of their medical and dentistry curriculum in English. At UPMS, English is one of three official languages of instruction (alongside Hungarian and German), and most medical and dentistry students are fluent in English. Consequently, language did not pose a barrier to participation in the survey. The questionnaire was adapted from a validated instrument developed by Malmström et al. [4] for assessing students’ attitudes toward AI in education. The final version (see Supplementary material 1) contained six demographic questions, 16 mandatory Likert-scale items (six-point scale), and four open-ended items exploring attitudes toward AI, familiarity and use of AI tools, perceived benefits, challenges, and ethical considerations.

The survey was pretested in September 2024 with four students (three international and one Hungarian) to ensure clarity and relevance. Minor revisions were implemented based on their feedback. Participation was voluntary, and respondents were informed about their right to withdraw at any time. An introductory page detailed study aims, data protection, and consent requirements, followed by a mandatory consent statement:

“By proceeding with this questionnaire, you confirm that: (1) your participation is voluntary, (2) your responses will remain anonymous, and (3) you consent to the use of your anonymous data for research or educational purposes.” The survey took approximately 10–15 min to complete.

### Qualitative data collection

In addition to the open-ended survey items, nine semi-structured, one-to-one interviews were conducted with educators and practising physicians between October 1 and October 22, 2024. They were invited through professional networks and direct email contact based on their involvement in medical education or clinical teaching. Participation was voluntary. The group included four educators from UPMS, Hungary, two educators from ONH, Norway, and three practising physicians from both Hungary and Norway. Participants represented a range of medical and academic disciplines, including general practice, gastroenterology, surgery, biochemistry, medical communication, behavioural sciences, cell biology, dentistry and pathophysiology. The interview participants received the information sheet in advance and provided informed consent prior to the interviews. All participants were informed of their right to withdraw at any time without consequences, and all data were anonymized prior to analysis.

The interviews were conducted, recorded digitally, and transcribed verbatim. The interview guide (see Supplementary material 2) comprised 20 open-ended questions addressing perceptions of AI, pedagogical and clinical implementation, ethical considerations, and future directions. The guide was pretested in September 2024 with one Hungarian and one Norwegian educators, leading to minor refinements. Interviews were conducted in English or Norwegian, depending on participant preference. Norwegian responses were translated into English by the second author, who is fluent in both languages. Interviews lasted between 20 and 40 min.

### Sample sufficiency and thematic saturation

Interview recruitment continued until thematic saturation was reached, meaning that additional interviews were no longer yielding new themes or substantial insights. After conducting nine interviews with educators (*n* = 6) and practising physicians (*n* = 3), the research team determined that saturation had been achieved. This sample was therefore considered sufficient to capture the range of perspectives on AI in medical education among experienced educators and practising physicians.

### Data analysis

Quantitative data were analysed using descriptive statistics, including frequencies, percentages, and mean values. Comparisons between medical and dental students were conducted using independent-samples t-tests to examine group differences across individual questionnaire items assessing attitudes toward AI in medical education. Prior to hypothesis testing, assumptions of normality and homogeneity of variances were evaluated. Homogeneity of variances was assessed using Levene’s test. When the assumption of equal variances was met, standard independent-samples t-tests were applied. In cases where Levene’s test indicated unequal variances (*p* < .05), Welch’s t-test (unequal variances t-test) was used to provide a more robust estimate of group differences. All tests were two-tailed, and statistical significance was set at *p* < .05. Although the group sizes were unequal, independent-samples t-tests are considered robust to such imbalance. However, the smaller sample size of dental students may have reduced statistical power for detecting small effect sizes.

Qualitative data were examined thematically, following the Braun and Clarke [13] framework. Interview transcripts were coded manually by the first author through iterative categorization and theme development. To enhance analytic rigor, emerging codes and themes were discussed with the second author, and interpretations were refined until consensus was reached. Themes derived from the interviews were compared with the survey findings to identify convergences and divergences. Findings from both datasets were then interpreted in conjunction with relevant literature on AI in medical and dentistry education to strengthen validity and situate the results within the broader academic context.

### Mixed-methods integration and triangulation

Triangulation was conducted at the interpretation stage by systematically comparing quantitative survey results with themes derived from the qualitative interviews. Areas of convergence were used to strengthen confidence in the findings, while divergences and apparent discrepancies were examined in greater depth to explore contextual or role-specific explanations. Discrepancies between datasets were not treated as contradictions but as analytically informative, prompting iterative discussion among the research team and interpretation in relation to existing literature on AI in medical and dentistry education. This integrative approach allowed the quantitative data to provide breadth and the qualitative data to offer depth and contextualization.

### Ethical considerations

The study was conducted in accordance with the Declaration of Helsinki. Ethical approval was obtained from the Regional Research Ethics Committee of UPMS (Ref. No. 9909-PTE 2024). ONH confirmed that additional local approval was not required, as institutional collaboration was covered under the UPMS approval. All participants provided informed consent. Confidentiality and anonymity were maintained throughout, and all data were stored securely in accordance with institutional and international data protection standards.

## Results

### Quantitative findings

#### Demographics

As detailed in Table 1 below, a total of 344 medical and dentistry students completed the survey. The majority, 66.9% (230/344), were female and most, 88.1% (303/344), were enrolled at the University of Pécs Medical School (UPMS). A smaller subset, 11.9% (41/344), represented Oslo New University College (ONH). At ONH, the response rate was high (41/100), though the absolute number remained lower than at UPMS. Because of this imbalance, no statistical comparisons between the two groups were conducted. Instead, all responses were analyzed together as a pooled dataset. Participants represented all years of study, with a predominance of students in the first to third years of training.

**Table 1:** Demographic characteristics of students (*n* = 344)

Demographic variable	*n* (%)
Gender	
Female	230 (66.9)
Male	113 (32.8)
Prefer not to say	1 (0.3)
Nationality	
Norway	156 (45.3)
Hungary	82 (23.8)
Germany	27 (7.8)
Iran	11 (3.2)
Nigeria	8 (2.3)
Other	60 (17.4)
University	
UPMS	303 (88.1)
ONH	41 (11.9)
Field of study	
Medicine	303 (88.1)
Dentistry	41 (11.9)
Level of study	
Undergraduate student	337 (98.0)
PhD student	7 (2.0)

### Attitudes toward AI in medical and dentistry education

**Fig. 1:**
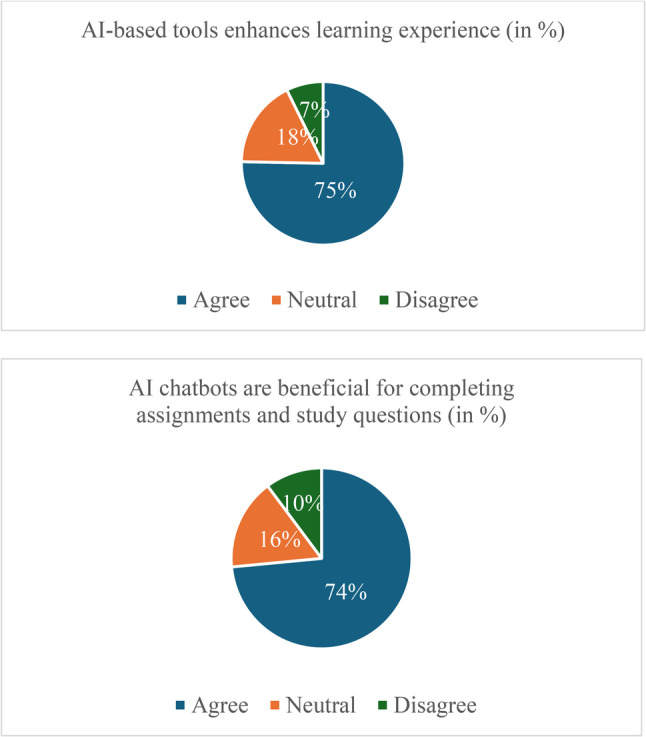
Student attitudes toward AI in medical and dentistry education (*n* = 344)

Most students expressed positive views regarding the integration of AI into their studies. As shown in Fig. 1, a total of 75.3% (259/344) agreed that AI-based tools enhanced their learning experience, including 30.8% (106/344) who strongly agreed and 44.5% (153/344) who agreed. Only 7.2% (25/344) disagreed, while 17.4% (60/344) remained neutral. Similarly, 73.5% (253/344) reported that AI chatbots were beneficial for completing assignments and study questions, suggesting broad enthusiasm for AI-assisted learning, although a minority remained sceptical.

### Familiarity and usage of AI tools

**Fig. 2:**
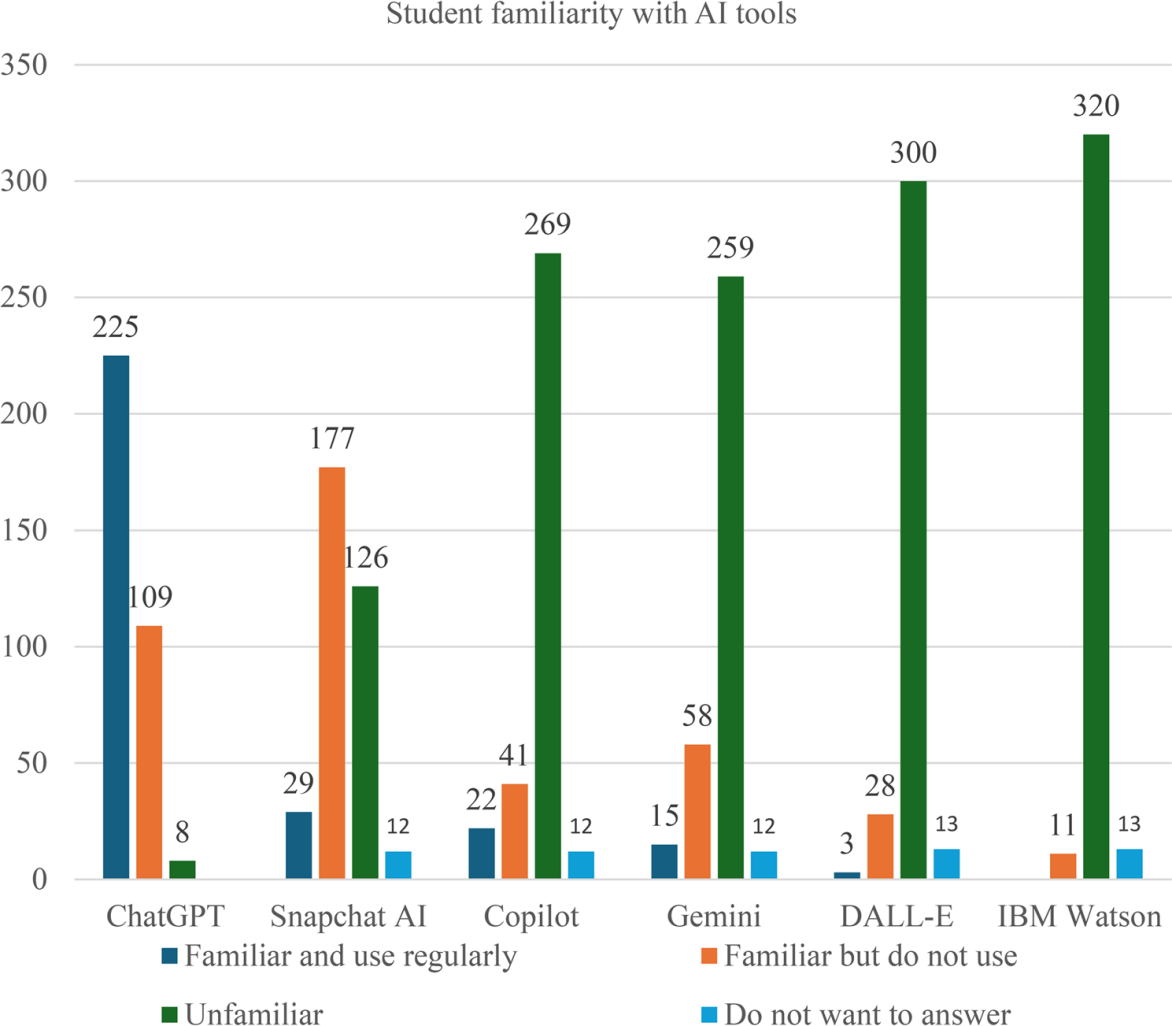
Level of familiarity with selected AI tools among medical and dentistry students (*n* = 344)

Awareness of generative AI tools was high, particularly for ChatGPT (Fig. 2). Nearly all respondents reported familiarity with ChatGPT and many used it regularly. Other tools such as Snapchat AI, Gemini, and Copilot were also recognized, while DALL-E and IBM Watson were less familiar. Despite this high awareness, most students reported using AI tools occasionally rather than daily, indicating a gap between recognition and consistent academic application.

### Perceived benefits of AI

**Fig. 3:**
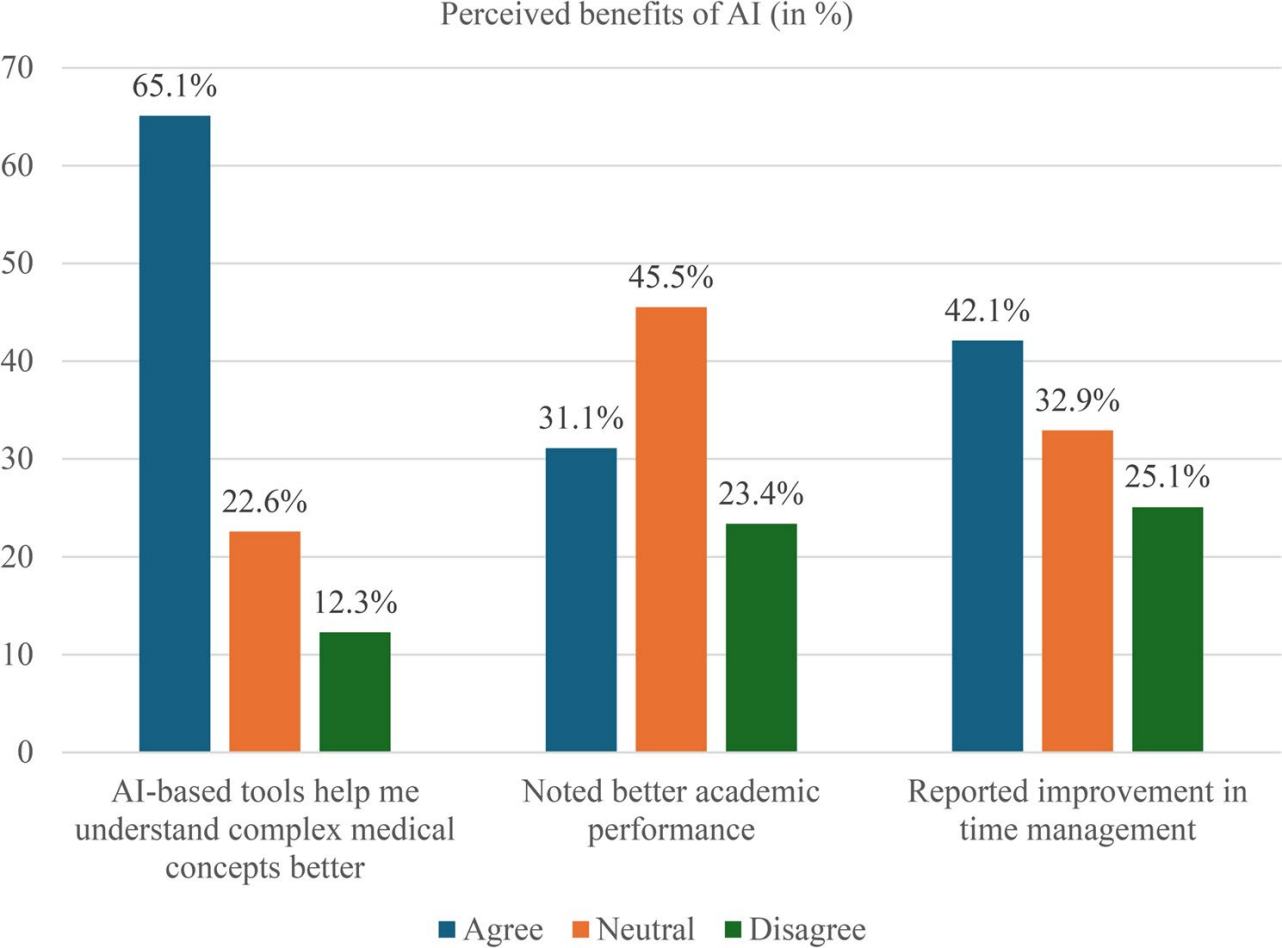
Reported educational benefits of AI use among medical and dentistry students (*n* = 344)

Students identified several benefits of using AI tools in their education, as detailed in Fig. 3. A total of 65.1% (224/344) agreed that AI improved their understanding of complex concepts, emphasizing its role as a supplement to traditional instruction. In addition, 42.1% (145/344) reported that AI tools enhanced their time management, likely due to faster access to information. However, only 31.1% (107/344) believed AI had improved their academic performance, and 32.0% (110/344) reported increased motivation to study. These results indicate that students perceive AI as helpful for efficiency and comprehension, though its direct impact on grades and motivation appears limited.

### Concerns and challenges

**Fig. 4:**
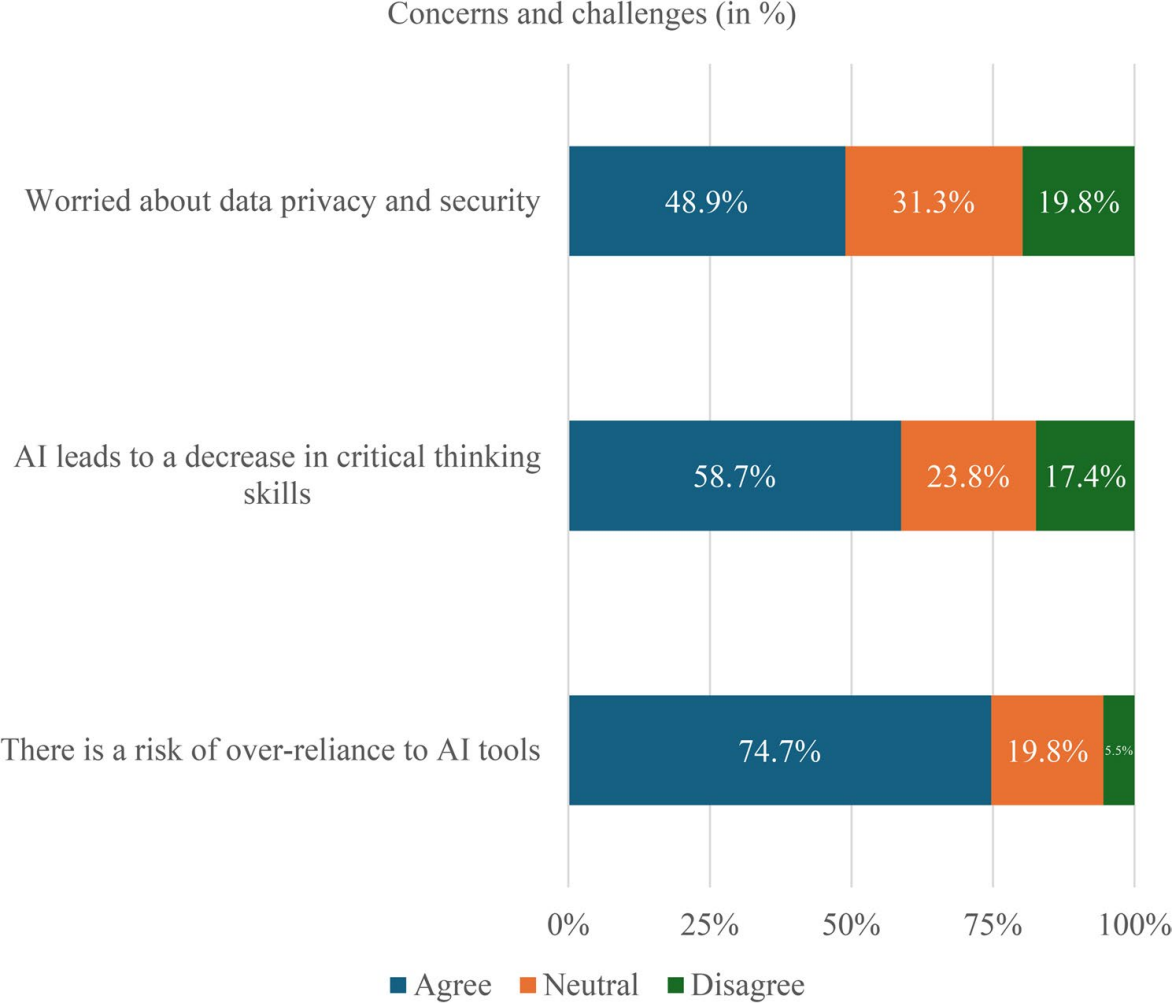
Student concerns regarding AI in medical and dentistry education (*n* = 344)

Despite overall optimism, several concerns were identified, as Fig. 4 demonstrates. Nearly half of the students, 48.9% (168/344) expressed worries about data privacy and security. More than half, 58.7% (202/344) were concerned that reliance on AI could reduce critical thinking, while 74.7% (257/344) acknowledged the risk of over-reliance undermining independent learning. Furthermore, 75.3% (259/344) reported difficulty fully trusting the accuracy of AI-generated information. These findings indicate that students recognize both the advantages and limitations of AI and approach its use with caution.

### Ethical considerations

Many students highlighted the importance of clear ethical guidelines for AI use. A majority, 56.7% (195/344) agreed that stricter regulations for AI in education were necessary, and 57.8% (199/344) emphasized the need to establish ethical frameworks to prevent misuse. These results suggest that students view AI as a valuable tool but recognize that its responsible integration requires explicit institutional policies to safeguard academic integrity.

### Comparative analysis of medical and dental students’ attitudes toward AI

Overall, the findings indicate that medical and dental students hold largely similar attitudes toward the use of AI, with most dimensions showing no statistically significant differences between the two groups. This suggests a broadly shared acceptance of AI tools across health professions education. However, statistically significant differences emerged in three dimensions: perceived benefits of AI, privacy concerns, and perceived risk of over-reliance on AI, highlighting profession-specific sensitivities (Table 2).

**Table 2:**
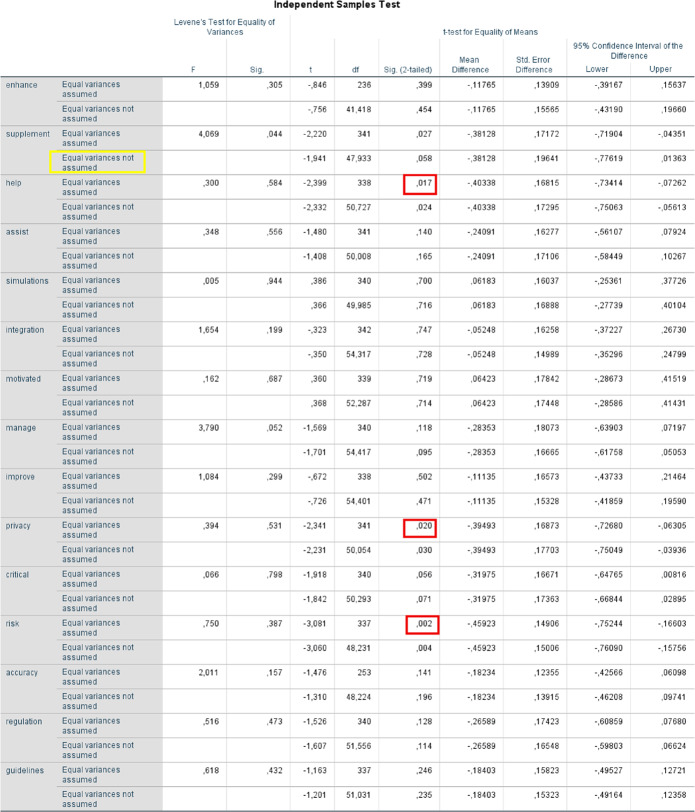
Independent Samples Test (see Supplementary material 3 for a more detailed version)

### Perceived benefits of AI

A statistically significant difference was observed for the item “AI-based tools help me understand complex medical concepts better” (*p* = .017), with medical students reporting higher levels of agreement than dental students. This finding suggests that medical students perceive AI tools as more beneficial for supporting the comprehension of complex theoretical material, potentially reflecting differences in curricular structure, content density, or patterns of AI use between the two programmes.

### Privacy and data security concerns

Medical students also expressed significantly greater concern regarding privacy and data security (*p* = .020) in response to the item “I am concerned about the privacy and security of my data when using AI-based tools.” This may indicate heightened awareness among medical students of data protection issues, possibly stemming from more frequent exposure to clinical environments, institutional regulations, or discussions surrounding patient data confidentiality.

### Perceived risk of over-reliance on AI

The strongest group difference was found for the item “There is a risk of over-reliance on AI tools among students” (*p* = .002), with medical students again reporting higher agreement. This result suggests a greater sensitivity among medical students to the potential negative consequences of excessive AI use, particularly with regard to clinical reasoning, independent decision-making, and professional competence development.

### Assumption testing and variance adjustment

For the item “AI chatbots are an effective supplement to traditional teaching methods,” Levene’s test for equality of variances was statistically significant (*p* = .044), indicating a violation of the homogeneity of variance assumption. Consequently, a Welch-adjusted independent-samples t-test was applied. Under this more conservative test, the difference between medical and dental students did not reach statistical significance (*p* = .058). While medical students tended to rate AI chatbots more favourably as a supplement to traditional teaching, this result should be interpreted as a non-significant trend rather than a confirmed group difference.

### Summary of comparative findings

In summary, statistically significant differences between medical and dental students were confined to perceived benefits of AI, privacy concerns, and the risk of over-reliance on AI, with medical students consistently reporting higher levels of agreement. Most other dimensions reflected broadly similar attitudes toward AI integration across the two groups. Although the sample included a substantially larger number of medical students than dental students, independent-samples tests are generally robust to unequal group sizes. Nevertheless, the smaller dental student sample may have limited statistical power to detect smaller effects, and this should be considered when interpreting non-significant findings.

### Qualitative findings

In addition to the open-ended questions in the online survey, nine semi-structured interviews were conducted with educators (*n* = 6) and practising physicians (*n* = 3) from UPMS and ONH, each with 10–20 years of teaching or clinical experience. Four main themes emerged from the thematic analysis: *enhanced learning potential*, *concerns about over-reliance*, *ethical and regulatory needs*, and *diverse perspectives on clinical applicability*. To increase theoretical transparency, the qualitative themes are presented with their primary alignment to the extended Technological Pedagogical Content Knowledge (TPACK) framework.

#### Enhanced learning potential (Technological Knowledge – TK)

Both educators and physicians emphasized AI’s capacity to enrich the learning process. Participants noted that AI-based simulations and chatbots can provide students with a deeper understanding of theoretical knowledge. One educators (E1) from UPMS remarked:

“AI has the potential to revolutionize medical education, but it must remain a supplement, not a substitute, for teaching.”

Similarly, a physician (P1) explained:

“It allows students to test themselves and get explanations instantly, which is something traditional textbooks cannot offer.”

Open-ended student responses reflected comparable enthusiasm for AI’s learning benefits. One student (S1) wrote:

“AI explained respiratory acidosis in a way that no teacher or YouTube video was able to, I fully understand the concept to this day.”

Another (S2) commented:

“There are many subjects I passed with the help of AI. It gives me good explanations when I ask the right questions and helps me understand rather than memorize everything.”

Students also described improved efficiency and engagement, for example (S3):

“Miscalculated the time before an exam, so I used ChatGPT to make summaries, it helped me get through the material and pass.”

Overall, these responses illustrate that students and educators alike perceive AI as a valuable supplement that enhances comprehension and efficiency.

#### Concerns about over-reliance (Pedagogical Knowledge – PK)

A recurring theme was apprehension that excessive dependence on AI might hinder independent thinking. Seven of the nine interviewees mentioned this issue, warning that constant access to ready-made answers could impede the development of analytical and clinical reasoning skills. An educator (E2) at ONH explained:

“If students come to rely on AI for every answer, they risk losing the ability to reason clinically and solve problems independently.”

An educator from UPMS (E3) expressed a similar view:

“AI can make students passive consumers of information instead of active learners; they need to learn how to think, not just what to ask.”

Another educator (E4) added:

“When students trust AI too much, they stop questioning its accuracy, which can be dangerous in clinical reasoning.”

Students shared related experiences in the open-ended survey responses. One admitted (S4):

“Sometimes when I ask AI if the answer it provided is correct, it changes its answer, it forces me to double-check and wastes time.”

Another wrote (S5):

“It’s easy to get lazy with AI. Instead of thinking, I just ask it to explain things again until it makes sense.”

Interestingly, these concerns mirrored the broader patterns observed in the survey data.

#### Ethical and regulatory needs (Contextual Knowledge – XK)

Educators and physicians consistently stressed the importance of establishing ethical guidelines for AI use in education. Transparency, data protection, and responsible practice were among the main concerns. An educator from ONH (E5) stated:

“Ethical guidelines are crucial to prevent misuse and ensure responsible use of AI tools.”

A Hungarian educator (E6) echoed this need:

“We need institutional rules for how AI should be used; without them, students won’t know what counts as acceptable or plagiarism.”

Another educator (E3) observed:

“There must be a clear line between using AI for learning and using it to replace your own work; otherwise, it risks undermining academic integrity.”

Students expressed similar ethical concerns in the survey. One wrote (S6):

“AI is great for studying, but sometimes it gives too much help, it’s hard to know where to draw the line.”

Another suggested (S7):

“We should have clearer university policies on how we can use AI because everyone uses it differently, some responsibly, some not.”

In summary, these views demonstrate broad agreement among faculty and students that ethical frameworks and institutional policies are essential to maintain academic honesty and professionalism.

#### Diverse perspectives on clinical applicability (Content Knowledge – CK; Pedagogical Knowledge – PK)

While participants recognized AI’s value for theoretical and conceptual learning, they were cautious about its use in clinical training. Educators generally agreed that AI tools can clarify complex biomedical topics but doubted their relevance for bedside teaching. One physician summarized (P2):

“AI can explain physiology, but it cannot teach empathy or clinical judgment.”

Another physician added (P3):

“AI might simulate patient cases, but it cannot replicate the nuances of real patient interactions.”

An educator from ONH emphasized (E4):

“Medical education must remain patient-centred. AI should help students prepare, not replace real clinical exposure.”

Students also recognized these limitations. One explained (S5):

“AI is great for theory, but during hospital practice it’s useless. Patients don’t behave like algorithms.”

Another reflected (S7):

“AI can summarize diseases, but it doesn’t teach you how to talk to patients or manage uncertainty.”

These insights indicate consensus that AI should play a complementary rather than substitutive role in medical training. Both faculty and students valued its theoretical utility but emphasized that essential clinical competencies, empathy, communication, and judgment, must be developed through real-world experience.

### Comparative perspectives of educators and practising physicians

Although educators and practising physicians shared broadly similar views on the role of AI in medical education, a closer comparative analysis revealed subtle but meaningful discipline-specific emphases across the four themes.

Regarding enhanced learning potential, both groups perceived AI as a valuable supplementary educational tool, however, their focus differed. Educators primarily emphasized AI’s role in supporting conceptual understanding, structured learning, and curriculum-aligned knowledge acquisition, often framing AI as a pedagogical aid that must remain carefully integrated into formal teaching. In contrast, practising physicians highlighted AI’s value for self-directed learning and rapid clarification, particularly appreciating its immediacy and responsiveness compared to traditional resources. This distinction suggests that educators view AI through a curricular and instructional lens, whereas practising physicians approach it from a pragmatic, learner-centred perspective shaped by clinical time constraints.

Differences were also evident in concerns about over-reliance. Educators more frequently articulated worries about the erosion of critical thinking, and active learning habits, expressing concerns that AI may foster passivity and uncritical acceptance of information. Practising physicians, while acknowledging similar risks, framed over-reliance more in terms of clinical safety and reasoning, emphasizing the danger of students trusting AI outputs without sufficient contextual judgment. These differing emphases reflect educators’ responsibility for cultivating learning processes, while practising physicians prioritize the downstream implications for patient care.

In the theme of ethical and regulatory needs, both groups strongly agreed on the necessity of institutional guidelines. However, educators tended to focus on academic integrity, assessment validity, and plagiarism, whereas practising physicians emphasized professional accountability, data protection, GDPR issues and the ethical implications of AI-informed decision-making in healthcare contexts. This divergence highlights how professional roles shape ethical priorities: educators are primarily concerned with maintaining educational standards, while practising physicians extend ethical considerations to the broader healthcare system.

The most pronounced contrast emerged in perspectives on clinical applicability. Practising physicians were more sceptical of AI’s usefulness in developing bedside skills, clinical judgment, and patient communication, repeatedly stressing that empathy, uncertainty management, and situational awareness cannot be meaningfully replicated by AI systems. Educators, while sharing these reservations, were somewhat more open to AI’s role in pre-clinical preparation, such as case simulations and conceptual rehearsal, provided that real patient interaction remains central. This suggests that practising physicians’ day-to-day engagement with patient care heightens their sensitivity to the limitations of AI in authentic clinical encounters.

Overall, these comparative findings indicate that while educators and practising physicians largely converge in their appraisal of AI as a complementary tool, their disciplinary roles shape distinct priorities, concerns, and thresholds of acceptance. Recognizing these differences is essential for designing AI-integrated medical curricula that are pedagogically sound, clinically grounded, and ethically robust.

### Summary of findings

Integrating the quantitative and qualitative findings reveals a high degree of convergence across participant groups, alongside meaningful differences in emphasis shaped by professional roles. Across both datasets, AI was consistently perceived as a valuable supplementary tool that enhances learning efficiency, supports conceptual understanding, and facilitates self-directed study. At the same time, concerns regarding over-reliance and the potential erosion of critical and clinical reasoning emerged strongly in both student survey responses and educator and clinician interviews, underscoring shared caution toward uncritical AI use.

Clear divergence was observed in how ethical and professional implications were framed. Students predominantly viewed AI through a pragmatic, learning-oriented lens, focusing on immediate benefits for studying and assessment preparation, whereas educators and practising physicians emphasized academic integrity, professional responsibility, data protection, and downstream implications for patient care. Similarly, while all groups acknowledged AI’s usefulness for theoretical learning, faculty and practising physicians were more sceptical of its applicability to bedside teaching, highlighting the irreplaceable role of real-world clinical experience, communication, and empathy. These convergent and divergent patterns across data sources are summarized in Table 3, which provides a joint display of key quantitative and qualitative findings.

**Table 3:** Summary of the key findings of both the quantitative and qualitative results, highlighting the international, multi-stakeholder perspective

	Quantitative results	Qualitative results	Interpretation
	Students (*n* = 344)	Educators and Physicians (*n* = 9)	
Perceived Learning Benefits	75.3% reported improved learning experiences; AI clarified complex concepts and enhanced efficiency	Noted AI’s potential to supplement teaching with simulations, explanations, and self-testing	Consensus that AI enhances learning, but not a replacement for traditional methods
Over-Reliance / Critical Thinking Concerns	58.7% worried AI may reduce critical thinking	Highlighted risks of dependence undermining clinical reasoning	Caution increases with professional experience; need for structured integration
Privacy and Accuracy	48.9% cited data security concerns	Emphasized transparency, regulation, and responsible AI use	Ethical and regulatory safeguards are essential for safe adoption
Practical vs. Professional Focus	AI framed as immediate, pragmatic aid (efficiency, conceptual understanding)	Emphasized long-term professional implications (ethics, academic integrity, clinical judgment)	Divergence reflects different roles and responsibilities; importance of addressing both perspectives
Ethics and Regulation	Limited student awareness of ethical implications	Strong emphasis on regulation, clinical integrity, and professional standards	Guidance and structured policies required to ensure responsible AI integration
Overall Attitude	Enthusiastic about AI benefits but cautious	Supportive yet cautious; stress professional identity and patient care	Integration should be deliberate, structured, and ethically grounded across international contexts

In summary, these findings indicate that while AI integration in medical and dentistry education is broadly welcomed, its effective implementation requires structured pedagogical guidance, explicit ethical frameworks, and clear boundaries that preserve critical thinking, professional identity, and patient-centred care across diverse international contexts.

## Discussion

This study explored the perspectives of international medical and dentistry students, educators, and physicians regarding the integration of artificial intelligence into medical and dentistry education. The findings indicate generally positive attitudes, with most students reporting that AI tools enhanced learning, improved efficiency, and clarified complex concepts. At the same time, both students and faculty expressed concerns about data privacy, over-reliance, and the potential erosion of critical thinking. Educators and physicians emphasized these concerns more strongly, highlighting the importance of ethical oversight and questioning AI’s applicability to bedside teaching. Triangulating these perspectives reveals a shared view that AI serves as a valuable supplement to medical and dentistry education but should be implemented cautiously and responsibly.

In addition to these overarching trends, comparative quantitative analyses revealed subtle but meaningful differences between medical and dental students. While attitudes toward AI were largely similar across the two groups, medical students reported significantly stronger agreement regarding AI’s role in supporting the understanding of complex concepts, as well as heightened concerns about data privacy and the risk of over-reliance on AI tools. These findings suggest that although acceptance of AI is broadly shared across health professions education, its perceived benefits and risks are shaped by professional context and curricular demands. Azer et al. [14] emphasized that medical schools worldwide face similar challenges, curriculum alignment, assessment redesign, and regulatory preparedness when introducing AI into teaching. Our findings align with large-scale student surveys showing widespread enthusiasm for AI in higher education. In Sweden, 95% of university students reported familiarity with ChatGPT, and more than half believed it improved their learning effectiveness [4]. Similarly, in a multi-country study of health profession students, 77.6% supported integrating AI into curricula, although 66.4% demonstrated limited knowledge of AI principles [3]. Comparable findings were also reported among Chinese medical students, where high awareness of AI contrasted with limited pedagogical understanding and inconsistent daily use [15]. In the present study, 75.3% of students agreed that AI enhanced learning, consistent with these international trends. However, challenges about privacy, accuracy, and ethics echoed findings from other educational settings [1, 14, 15]. Concerns regarding critical thinking and the humanistic aspects of medicine also parallel prior work. In India, 69.2% of medical students worried that AI would reduce the humanistic dimension of care, and more than half feared negative effects on the patient–physician relationship [6]. Similarly, participants in the current study expressed concern that dependence on AI could undermine the development of clinical reasoning and independent judgment. Notably, this concern was more pronounced among medical students than dental students, indicating a heightened sensitivity to the potential impact of AI on clinical reasoning within medical training contexts.

These observations align with broader reviews noting that uncritical AI adoption risks diminishing deep learning and problem-solving skills [1, 16]. The strong call for ethical regulation observed in this study is likewise reflected in existing literature. Issa et al. [3] also found that most health profession students considered regulation essential for effective AI integration. Lye and Lim [7] emphasized the need for assessment redesign to uphold academic integrity in the context of generative AI, while Kurtz et al. [2] highlighted the importance of institutional policy and faculty training for responsible adoption. Collectively, these findings suggest that while the educational potential of AI is widely acknowledged, stakeholders consistently demand structured guidance to prevent misuse and preserve integrity. Beyond these broad consistencies, this study revealed distinct differences between stakeholder groups. These qualitative differences align with the quantitative findings, where students emphasized immediate learning benefits, while educators and physicians foregrounded ethical risks, professional responsibility, and long-term consequences for clinical practice. Students primarily viewed AI as a practical study aid, emphasizing efficiency, accessibility, and comprehension. Educators and physicians, in contrast, focused on professional identity, ethical responsibility, and the preservation of clinical judgment. These differences likely reflect varying priorities: students seek immediate academic support, while professionals remain attentive to long-term implications for patient care and medical and dentistry standards. Similar patterns have been reported in broader analyses, where optimism about AI use is typically strongest among students and more reserved among senior educators and practising physicians [1].

The four themes identified in the qualitative analysis can be directly aligned with the extended Technological Pedagogical Content Knowledge (TPACK) framework. Students’ emphasis on efficiency, accessibility, and comprehension predominantly mapped onto Technological Knowledge (TK), whereas educators’ and physicians’ concerns regarding over-reliance, erosion of critical thinking, and ethical responsibility reflected Pedagogical (PK) and Contextual Knowledge (XK). Perceived benefits related to efficiency, clarification of complex concepts, and self-directed learning primarily reflect Technological Knowledge (TK), particularly in relation to generative AI tools. Concerns about over-reliance, passive learning, and diminished critical thinking correspond to Pedagogical Knowledge (PK), emphasizing the need for instructional strategies that actively scaffold reasoning rather than replace it. Participants’ confidence in disciplinary content, alongside scepticism toward AI’s role in bedside teaching, highlights the continued centrality of Content Knowledge (CK) and its limits when mediated by technology. Finally, strong emphasis on ethical regulation, data privacy, professional responsibility, and long-term implications for patient care aligns with Contextual Knowledge (XK), underscoring the relevance of the expanded TPACK model in the context of generative AI. Across stakeholder groups, participants displayed well-established content and pedagogical knowledge (CK and PK) but more limited technological and contextual knowledge (TK and XK). This suggests that current training does not yet fully equip medical and dentistry educators and students to address the ethical and evaluative challenges posed by generative AI. The findings therefore support Mishra et al.’s [11] argument that effective AI integration requires extending TPACK to include contextual awareness, critical appraisal of AI outputs, and ethical reflection. Therefore, the authors believe this study provides empirical support for the expanded TPACK model within medical and dentistry education.

In summary, our study demonstrates that while enthusiasm for AI in medical and dentistry education is strong among international medical and dentistry students, educators and physicians highlight the risks of over-reliance, ethical challenges, and the importance of preserving clinical reasoning. Integration should therefore be guided by structured curricula, ethical safeguards, and a clear emphasis on positioning AI as a supplement to, rather than a replacement for traditional medical teaching.

### Implications for medical and dentistry education

The findings suggest that AI can play a constructive role in medical and dentistry education when implemented deliberately. First, curricula should integrate AI literacy to help students not only use AI tools effectively but also critically evaluate their outputs. Second, faculty development programs are necessary to build educators’ confidence and competence in guiding students’ AI use. Third, institutions and professional bodies should establish ethical and regulatory frameworks to ensure responsible implementation and protect data security. Importantly, AI should complement, not replace hands-on clinical teaching, preserving the development of clinical reasoning, empathy, and communication skills. Future studies could extend this work by examining AI integration longitudinally, tracking how perceptions evolve as AI becomes embedded in medical and dentistry curricula. Feigerlova et al. [17] (2025) found in their systematic review that AI-enhanced learning tools improved knowledge, skills, and engagement but highlighted the need for long-term evaluations to confirm sustained benefits. Addressing this gap through intervention studies, testing AI-based modules, clinical simulations, or ethics frameworks would yield practical evidence for best practices. Expanding stakeholder inclusion to encompass patients, policymakers, and curriculum developers would further enrich understanding of AI’s impact on medical and dentistry education and professional formation.

### Limitations

Some limitations should be acknowledged in this study. Although participants were drawn from two distinct European contexts, including a large cohort of international students, the findings may not fully capture global perspectives on the examined phenomena. The use of online self-report surveys may have introduced response biases, such as over-reporting of socially desirable attitudes toward AI or under-reporting of challenges experienced. Convenience sampling may have favored students and educators with a particular interest in AI, potentially limiting the representativeness of the findings. Institutional differences between UPMS and ONH, including curriculum design, language of instruction, and teaching practices, may have influenced participants’ perceptions and contributed to variation in responses. In addition, the uneven distribution of respondents between UPMS and ONH means that the results primarily reflect the perspectives of students at UPMS, which limits generalizability to ONH or other institutional contexts. As the study used a cross-sectional design, the results represent participants’ perceptions at a single point in time. Future research incorporating larger, more balanced, and multi-institutional samples could provide a more comprehensive and generalizable understanding of these issues.

## Conclusion

This cross-institutional, mixed-methods study provides a comprehensive exploration of the evolving role of artificial intelligence (AI) integration in medical and dentistry education from the perspectives of international medical and dentistry students, educators, and practising physicians. By incorporating comparative analyses across health professions and stakeholder groups, the study demonstrates that while acceptance of AI is widespread, perceptions of its benefits and risks vary systematically according to professional role and educational context. AI is increasingly recognized as a potentially transformative tool in medical and dentistry education, particularly by students, while educators and practising physicians highlight its limitations and emphasize the need for structured, ethical, and context-sensitive implementation. Despite differences in emphasis among stakeholder groups, there was consensus that AI should complement rather than replace traditional teaching and clinical training. Effective integration of AI into medical and dentistry education requires a balanced approach that incorporates AI literacy for students, targeted training for educators, and the establishment of ethical frameworks to ensure responsible use. The identified differences between medical and dental students, alongside contrasting perspectives of students, educators, and physicians, underscore the need for differentiated and context-sensitive approaches to AI integration rather than uniform implementation across health professions education. By addressing these priorities, AI can serve as a valuable supplement to medical and dentistry training, supporting the next generation of physicians and dentists while preserving the core humanistic competencies of professionalism, empathy, and clinical judgment.

## Supplementary Information

Supplementary Material 1.

Supplementary Material 2.

Supplementary Material 3.

## Data Availability

The questionnaire, the semi-structured interview guide, and statistical tables used in this study are available in the Supplementary Materials. De-identified excerpts from qualitative responses are included in the article. Anonymized datasets generated and/or analysed during the study are available from the corresponding author on reasonable request.

## Abbreviations

AI: Artificial Intelligence
UPMS: University of Pécs Medical School
ONH: Oslo Nye Høyskole/Oslo New University College
TPACK: Technological Pedagogical Content Knowledge
