# Supplementary material for: AI in medical and dentistry education: perspectives from international students, educators and physicians
Source: BMC Med Educ. 2026 Feb 25;26:534. doi: 10.1186/s12909-026-08886-5 (PMC13040955; doi:10.1186/s12909-026-08886-5)
Supplement: Supplementary file 1 — Supplementary Material 1. [file 12909_2026_8886_MOESM1_ESM.pdf]

Supplementary Material 1: Questionnaire

1. What is your gender? (Male, Female, Non-binary, Prefer not to say)
2. Which university do you attend? (Medical School, University of Pécs, ONH, Oslo, Other)
3. What is/will be your field of study? (Medicine, Dentistry)
4. What is your level of study? (open answer)
5. What is your current year of study? (open answer)
6. What is your nationality? (open answer)

The following questions were Likert-scaled. Questions ranged as follows:

- Strongly disagree
- Disagree
- Neutral
- Agree
- Strongly Agree
- Do not want to answer

7. I believe AI-based tools enhance the learning experience in medical education.
8. Using AI chatbots to assist with assignments and study questions is beneficial.
9. AI chatbots are an effective supplement to traditional teaching methods.
10. AI-powered simulations and virtual labs provide a valuable supplement to hands-on clinical training.
11. The integration of AI in medical education is essential for keeping up with advancements in the healthcare field.
12. Familiarity with AI tools (each listed separately):
    - ChatGPT
    - DALL-E

- Copilot
- Gemini
- Perplexity
- AI Life
- IBM Watson
- Intercom
- Jasper
- Anthropic Claude
- Lyro
- Snapchat My AI
- Replika
- Chatsonic
- GPT UiO

13. AI-based tools help me understand complex medical concepts better.
14. I feel more motivated to study when using AI-based tools.
15. AI tools help me manage my study time more effectively.
16. Since using AI-based tools, I have noticed an improvement in my academic performance.
17. I am concerned about the privacy and security of my data when using AI-based tools.
18. The use of AI in education could lead to a decrease in critical thinking skills.
19. There is a risk of over-reliance on AI tools among students.
20. I sometimes find it difficult to trust the accuracy of information provided by AI tools.
21. AI should be regulated more strictly in educational contexts to prevent misuse.
22. I believe that ethical guidelines should be established for the use of AI in medical education.

The questionnaire included the following open-ended questions:

23. Can you share a story when you had a positive experience with the use of AI in medical education?
24. Can you share a story when you had a less positive experience with the use of AI in medical education?
25. Any lessons learned from these stories that you would like to share?
26. Any other comments or suggestions?
