# Supplementary material for: AI in medical and dentistry education: perspectives from international students, educators and physicians
Source: BMC Med Educ. 2026 Feb 25;26:534. doi: 10.1186/s12909-026-08886-5 (PMC13040955; doi:10.1186/s12909-026-08886-5)
Supplement: Supplementary file 2 — Supplementary Material 2. [file 12909_2026_8886_MOESM2_ESM.pdf]

Supplementary Material 2: Semi-structured interview questions

General Perception

1. What was your first thought when you learned about the rapid development and spread of AI?
2. Do you think AI has the potential to transform the medical education sector? If so, how?
3. How do you feel about the integration of AI in classroom/clinical settings?
4. What concerns, if any, do you have about the use of AI in education?
5. How informed do you feel about AI technology, and where do you typically get your information about it?

Usage and Implementation

6. Have you incorporated AI-based tools into your curriculum and teaching practices?
7. If yes, how have you ….
8. Did any of the tasks, presentations, assignments, etc. have to be changed?
9. Can you provide examples of specific AI applications or tools you use in your classes?
10. What challenges have you faced in implementing AI tools in your teaching?

Impact on Teaching

11. Can you describe your overall experience with integrating AI tools into your teaching methods?
12. What are your thoughts on the effectiveness of AI tools in enhancing medical education?
13. What do you think your students' relationship is with AI?
14. Do you think they want to use it to cheat or to make their lives easier?
15. How has the use of AI changed your approach to teaching medical students?
16. In what ways do you believe AI tools have improved or hindered student engagement and learning?

Ethical Considerations and Future Prospects

17. What ethical concerns do you have regarding the use of AI in medical education?
18. How do you see the role of AI evolving in medical education over the next five to ten years?
19. What support or resources would you need to better integrate AI into your teaching?
20. What support or resources would YOUR STUDENTS need to better understand and use AI?
