# Supplementary material for: AI in medical and dentistry education: perspectives from international students, educators and physicians
Source: BMC Med Educ. 2026 Feb 25;26:534. doi: 10.1186/s12909-026-08886-5 (PMC13040955; doi:10.1186/s12909-026-08886-5)
Supplement: Supplementary file 3 — Supplementary Material 3. [file 12909_2026_8886_MOESM3_ESM.pdf]

Independent Samples Test

		Levene's Test for Equality of Variances		t-test for Equality of Means					95% Confidence Interval of the Difference	
		F	Sig.	t	df	Sig. (2-tailed)	Mean Difference	Std. Error Difference	Lower	Upper
enhance	Equal variances assumed	1,059	,305	-,846	236	,399	-,11765	,13909	-,39167	,15637
	Equal variances not assumed			-,756	41,418	,454	-,11765	,15565	-,43190	,19660
supplement	Equal variances assumed	4,069	,044	-2,220	341	,027	-,38128	,17172	-,71904	-,04351
	Equal variances not assumed			-1,941	47,933	,058	-,38128	,19641	-,77619	,01363
help	Equal variances assumed	,300	,584	-2,399	338	,017	-,40338	,16815	-,73414	-,07262
	Equal variances not assumed			-2,332	50,727	,024	-,40338	,17295	-,75063	-,05613
assist	Equal variances assumed	,348	,556	-1,480	341	,140	-,24091	,16277	-,56107	,07924
	Equal variances not assumed			-1,408	50,008	,165	-,24091	,17106	-,58449	,10267
simulations	Equal variances assumed	,005	,944	,386	340	,700	,06183	,16037	-,25361	,37726
	Equal variances not assumed			,366	49,985	,716	,06183	,16888	-,27739	,40104
integration	Equal variances assumed	1,654	,199	-,323	342	,747	-,05248	,16258	-,37227	,26730
	Equal variances not assumed			-,350	54,317	,728	-,05248	,14989	-,35296	,24799
motivated	Equal variances assumed	,162	,687	,360	339	,719	,06423	,17842	-,28673	,41519
	Equal variances not assumed			,368	52,287	,714	,06423	,17448	-,28586	,41431
manage	Equal variances assumed	3,790	,052	-1,569	340	,118	-,28353	,18073	-,63903	,07197
	Equal variances not assumed			-1,701	54,417	,095	-,28353	,16665	-,61758	,05053
improve	Equal variances assumed	1,084	,299	-,672	338	,502	-,11135	,16573	-,43733	,21464
	Equal variances not assumed			-,726	54,401	,471	-,11135	,15328	-,41859	,19590
privacy	Equal variances assumed	,394	,531	-2,341	341	,020	-,39493	,16873	-,72680	-,06305
	Equal variances not assumed			-2,231	50,054	,030	-,39493	,17703	-,75049	-,03936
critical	Equal variances assumed	,066	,798	-1,918	340	,056	-,31975	,16671	-,64765	,00816
	Equal variances not assumed			-1,842	50,293	,071	-,31975	,17363	-,66844	,02895
risk	Equal variances assumed	,750	,387	-3,081	337	,002	-,45923	,14906	-,75244	-,16603
	Equal variances not assumed			-3,060	48,231	,004	-,45923	,15006	-,76090	-,15756
accuracy	Equal variances assumed	2,011	,157	-1,476	253	,141	-,18234	,12355	-,42566	,06098
	Equal variances not assumed			-1,310	48,224	,196	-,18234	,13915	-,46208	,09741
regulation	Equal variances assumed	,516	,473	-1,526	340	,128	-,26589	,17423	-,60859	,07680
	Equal variances not assumed			-1,607	51,556	,114	-,26589	,16548	-,59803	,06624
guidelines	Equal variances assumed	,618	,432	-1,163	337	,246	-,18403	,15823	-,49527	,12721
	Equal variances not assumed			-1,201	51,031	,235	-,18403	,15323	-,49164	,12358

Group Statistics

	What is/will be your field of study?	N	Mean	Std. Deviation	Std. Error Mean
enhance	1	34	3,4118	,85697	,14697
	2	204	3,5294	,73217	,05126
supplement	1	41	3,3902	1,20162	,18766
	2	302	3,7715	1,00701	,05795
help	1	41	3,3659	1,04298	,16289
	2	299	3,7692	1,00515	,05813
assist	1	41	3,6829	1,03535	,16169
	2	302	3,9238	,97006	,05582
simulations	1	41	3,8293	1,02231	,15966
	2	301	3,7674	,95520	,05506
integration	1	41	3,7561	,88827	,13872
	2	303	3,8086	,98817	,05677
motivated	1	41	3,0976	1,04415	,16307
	2	300	3,0333	1,07521	,06208
manage	1	41	2,9756	,98711	,15416
	2	301	3,2591	1,09816	,06330
improve	1	41	2,9756	,90796	,14180
	2	299	3,0870	1,00625	,05819
privacy	1	41	3,0488	1,07124	,16730
	2	302	3,4437	1,00587	,05788
critical	1	41	3,2683	1,04939	,16389
	2	301	3,5880	,99485	,05734
risk	1	39	3,5641	,88243	,14130
	2	300	4,0233	,87484	,05051
accuracy	1	39	3,4103	,81815	,13101
	2	216	3,5926	,68928	,04690
regulation	1	40	3,3500	,97534	,15421
	2	302	3,6159	1,04300	,06002
guidelines	1	40	3,5250	,90547	,14317
	2	299	3,7090	,94423	,05461

Abbreviations:

enhance: I believe AI-based tools enhance the learning experience in medical education

supplement: AI chatbots are an effective supplement to traditional teaching methods

help: AI-based tools help me understand complex medical concepts better

assist: Using AI chatbots to assist with assignments and study questions is beneficial

simulations: AI-powered simulations and virtual labs provide a valuable supplement to hands-on clinical training

integration: The integration of AI in medical education is essential for keeping up with advancements in the healthcare field

motivated: I feel more motivated to study when using AI-based tools

manage: AI tools help me manage my study time more effectively

improve: Since using AI-based tools, I have noticed an improvement in my academic performance

privacy: I am concerned about the privacy and security of my data when using AI-based tools

critical: The use of AI in education could lead to a decrease in critical thinking skills

risk: There is a risk of over-reliance on AI tools among students

accuracy: I sometimes find it difficult to trust the accuracy of information provided by AI tools

regulation: AI should be regulated more strictly in educational contexts to prevent misuse

guidelines: I believe that ethical guidelines should be established for the use of AI in medical education
